# Computational approaches to protein inference in shotgun proteomics

**DOI:** 10.1186/1471-2105-13-S16-S4

**Published:** 2012-11-05

**Authors:** Yong Fuga Li, Predrag Radivojac

**Affiliations:** 1 School of Informatics and Computing, Indiana University, Bloomington 150 S. Woodlawn Avenue, Bloomington, Indiana, 47405, USA

## Abstract

Shotgun proteomics has recently emerged as a powerful approach to characterizing proteomes in biological samples. Its overall objective is to identify the form and quantity of each protein in a high-throughput manner by coupling liquid chromatography with tandem mass spectrometry. As a consequence of its high throughput nature, shotgun proteomics faces challenges with respect to the analysis and interpretation of experimental data. Among such challenges, the identification of proteins present in a sample has been recognized as an important computational task. This task generally consists of (1) assigning experimental tandem mass spectra to peptides derived from a protein database, and (2) mapping assigned peptides to proteins and quantifying the confidence of identified proteins. Protein identification is fundamentally a statistical inference problem with a number of methods proposed to address its challenges. In this review we categorize current approaches into rule-based, combinatorial optimization and probabilistic inference techniques, and present them using integer programing and Bayesian inference frameworks. We also discuss the main challenges of protein identification and propose potential solutions with the goal of spurring innovative research in this area.

## Introduction

The main objective of mass spectrometry-based proteomics is to provide a molecular snapshot of the form (e.g. splice isoforms, post-translational modifications), abundance level, and functional aspects (e.g. protein-protein interactions, protein localization) of each protein in a biological sample [1-3]. Among proteomics strategies, bottom-up or shotgun proteomics has emerged as a high-throughput technology capable of characterizing hundreds of proteins at the same time. In this scenario, proteins in a sample are first digested into peptides, typically using site-specific proteolytic enzymes (e.g. trypsin). Peptides are then separated by liquid chromatography (LC) and analyzed by tandem mass-spectrometry (MS/MS) resulting in a set of MS/MS spectra [4]. In contrast to the top-down proteomics strategy, where intact proteins are directly analyzed through mass spectrometers, shotgun proteomics is characterized by high separation efficiency and mass spectral sensitivity. At the same time, it places higher demands on the computational and statistical techniques necessary for peptide identification, protein identification, and label-free quantification.

In a standard computational pipeline, MS/MS spectra from a mass spectrometer are searched against spectral libraries [5-8] and/or *in silico *spectra [9-14] corresponding to peptides from a protein database in order to provide *peptide-spectrum matches *(PSMs). Such a database search, depending on the parameters of the search and the MS/MS platform, can result in a large number of PSMs that are assigned scores indicating the confidence level of correct identification of the respective peptide. The next step is to assemble a list of *identified proteins *from all, or a subset of, PSMs and provide statistical confidence levels for each protein.

Protein identification is a special case of label-free protein quantification because, in an ideal scenario, each protein with a correctly inferred non-zero quantity (abundance) would be considered identified. However, label-free quantification has not yet reached the accuracy needed for the wide dynamic range of quantities observed in cellular or extracellular proteomes [15]. In addition, in many practical situations it suffices to only consider the existence of proteins in the sample and not their exact quantity. Thus, solving the more general and significantly more difficult problem of quantification to provide a solution to its subproblem may result in less accurate solutions to protein identification.

Obtaining a list of identified proteins from a set of peptide sequences with identification scores may seem straightforward. However, there are several factors that combine to challenge such intuition: (1) Usually only a small number of peptide identifications, mostly unreliable, are available for each protein [16]. This is because only the top-scoring PSMs for each peptide are typically included into the candidate set for peptide identifications, and among those candidates only a small subset are considered to be confident identifications. This leads to difficulties in providing confident protein identifications, e.g. if only a single peptide is identified from a protein. (2) Peptides, even those from the same protein, are not equally likely to be identified in a proteomics experiment [17-19]. The probability that a peptide is identified in a standard proteomics experiment has been referred to as *peptide detectability *[19], see additional file 1. (3) Many peptide sequences encountered in a typical proteomics workflow can be mapped to more than one protein in a database. These are referred to as *degenerate *or *shared peptides *[20,21]. It is a common situation that a eukaryotic sample contains more degenerate than *unique peptides*, i.e. peptides that can be mapped to only one protein. (4) It is non-trivial to estimate the false discovery rates (FDRs) of identified peptides and proteins. Some approaches to estimating peptide-level FDRs involve construction of decoy databases or use unsupervised estimation of class-conditional distributions (distributions of PSM scores given correct and false identifications, respectively). However, a large number of low-scoring PSMs may create difficulties in determining the certainty of both peptide and protein identification. While methods for the estimation of peptide-level FDRs have been well-characterized, computing protein-level FDRs remains an open problem [22,23].

The process of identifying proteins that are present in a biological sample is now widely framed as a statistical inference problem, and has been referred to as the *protein inference problem *[20,21]. To date, a number of approaches have been proposed to address this problem [20,35-37]. We categorize those approaches into three broad groups, noting that a particular method may exploit more than one strategy:

1. Rule-based strategies - methods that rely on a relatively small set of confidently identified (unique) peptides that are subsequently assigned to proteins.

2. Combinatorial optimization algorithms - methods that rely on constrained optimization formulations of the protein inference problem resulting, for example, in minimal protein lists that cover some or all confidently identified peptides.

3. Probabilistic inference algorithms - methods that formulate the problem probabilistically and assign identification probabilities for each protein in a database.

In the following sections, we provide justification for the development of advanced protein inference algorithms and then review the major computational strategies. All combinatorial optimization techniques are presented using a framework of integer programming; on the other hand, probabilistic algorithms are summarized using Bayesian inference principles. Our focus is also on the intuition behind the algorithms, the types of solutions generated, and the strengths and limitations of each method. We believe this information is essential in order to understand commonalities among the algorithms as well as their principal differences. It is also important for the proper interpretation of outputs from the various protein inference tools already applied in bottom-up proteomics.

### Notation

Before discussing algorithmic details, it is important to introduce notation that will be used throughout this paper. Let us consider a set of tandem mass spectra S from a proteomics experiment and let  be a database of proteins that the spectra are searched against. Let also  be the set of all peptides in the database and, similarly,  be the set of peptides that belong to protein $P_{i}$. We now define two sets of indicator variables as follows

$$t_{j}=\left\{\begin{array}{c} 1\\ 0 \end{array}\begin{array}{c} \text{if}\;\text{peptide}\;p_{j}\;\text{is}\;\text{confidently}\;\text{identified}\\ \text{otherwise} \end{array}\right)$$

and

$$x_{j}=\left\{\begin{array}{c} 1\\ 0 \end{array}\right)\begin{array}{c} \text{if}\;\text{peptide}\;p_{j}\;\text{is}\;\text{present}\;\text{in}\;\text{the}\;\text{sample}\\ \text{otherwise} \end{array}$$

Confident peptide identifications can be determined in several ways, typically by using strict FDR thresholds on the top-scoring PSMs (per peptide) and are estimated using a decoy database [22] or tools such as PeptideProphet [38], which calculate the posterior probability of a correct peptide identification. When posterior probabilities are available, stringent thresholds (e.g. 0.90) can be applied directly to those probabilities. Alternatively, sufficiently high scores from various search engines [9,39-42] are sometimes used to select confident identifications.

It is important to mention that variables $t_{j}$ and $x_{j}$ are different. For example, a peptide *p_j _*that is confidently identified, e.g. using an FDR threshold of 0.01, will result in setting $t_{j}=1$. On the other hand, $x_{j}$ can be seen as a hidden variable that is to be inferred. Accordingly,  refers to the probability that peptide  j is present in the sample given all the data from the mass spectrometer. A set of confidently identified peptides, using any of the above-mentioned approaches will be denoted as .

In some situations it will be necessary to consider peptides with explicit designations of their parent proteins. In those cases, the  j-th peptide derived from protein $P_{i}$ will be denoted as $p_{ij}$. Two or more such peptides will be allowed to have identical amino acid sequences. For example, peptides $p_{ij}$ and $p_{kl}$ ( i ≠  k) with identical amino acid sequences will be referred to as degenerate peptides. In the context of protein inference, peptides that occur multiple times only within a single protein will not be considered degenerate. Finally, we define

$$y_{i}=\left\{\begin{array}{c} 1\\ 0 \end{array}\begin{array}{c} \text{if}\;\text{protein}\;P_{i}\;\text{is}\;\text{present}\;\text{in}\;\text{the}\;\text{sample}\\ \text{otherwise} \end{array}\right)$$

Variable $y_{i}$ can be seen as an equivalent of $x_{j}$ at the protein level. Thus,  is the posterior probability that protein $P_{i}$ is present in the sample. The summary of notation and abbreviations is shown in Table 1.

**Table 1:** Summary of notation and abbreviations used throughout this paper.

Notation	Description
	Set of all fragmentation spectra outputted by mass spectrometer

	Set of spectra identified for peptide *j*

*s*	A single fragmentation spectrum,

$P_{i}$ or *i*	Protein *i*

*p_j _*or *j*	Peptide *j*

*p_ij_*	Peptide *j *derived from protein *i*; used to explicitly indicate the parent protein for peptide *j*

	Protein database, a set of proteins used for peptide and protein identification

	Peptide database, the set of all (tryptic) peptides derived from

	Set of peptides derived from protein $P_{i}$

$t_{j}$	Indicator variable, set to 1 if peptide is *p_j _*confidently identified

	Set of peptides that are confidently identified

*x_j_*	Indicator variable, set to 1 if is present in the sample

*y_i_*	Indicator variable, set to 1 if is present in the sample

*x *= (*x*_1_, ... , *x_j _*, ...)	Indicator vector representing all peptides in

*y *= (*y*_1_, ... , *y_i _*, ...)	Indicator vector representing all proteins in

*N*(*i*)	Set of peptides mapped to protein *P_i_*

*N*(*j*)	Set of proteins that contain peptide *p_j_*

*x*_*N*(*i*)_	Indicator vector representing peptides in

	Peptide identification probability, the probability that peptide *j *is present in the sample given the spectra identified for peptide *j*

*P *(*x_j _*= 1|*s*)	The probability of the PSM matching to be correct when peptide *j *is the top-scoring match of spectrum

	Protein posterior probabilities, the probability that protein *i *is present in the sample given all spectra

*d_ij _*(*q*)	Detectability of peptide *p_ij _*at some specified quantity *q*; effective detectability

	Detectability of peptide *p_ij _*at standard quantity *q*^0 ^; standard detectability

*d_ij_*	Detectability of peptide *p_ij_*; effective detectability

*NSP_ij_*	The estimated number of (identified) sibling peptides of peptide *p_ij_*, used by ProteinProphet to adjust the peptide identification probability

PSM	Peptide-spectrum match; when it is clear from the context, we use PSM to also refer to the top-scoring PSM per spectrum

FDR	False discovery rate; the fraction of incorrect peptide identifications in or the fraction of incorrect protein identifications in a given list outputted by a protein inference algorithm. FDR should be distinguished from the false positive rate (FPR), the fraction of all peptides (proteins) from the database that are not present in the sample but are predicted to be present (at a particular threshold).

## Protein inference: significance and algorithms

Our first goal is to investigate the influence of degenerate peptides and to show that their presence is often a major factor contributing to the challenges in protein inference. We analyze several cellular and serum samples and characterize the peptide identification process. The data include cell line and plasma samples from *Homo sapiens *[16], a tissue sample from *Mus musculus *[43], as well as samples from *Saccharomyces cerevisiae *[44] and *Deinococcus radiodurans *[24]. The sets of spectra were searched using MASCOT [39] against the human IPI database (v3.35), mouse IPI database (v3.35), Saccharomyces Genome Database (R63, 05-Jan-2010), and *D. radiodurans *proteins extracted from GenBank (27-Aug-2009), respectively.

**Figure 1:**
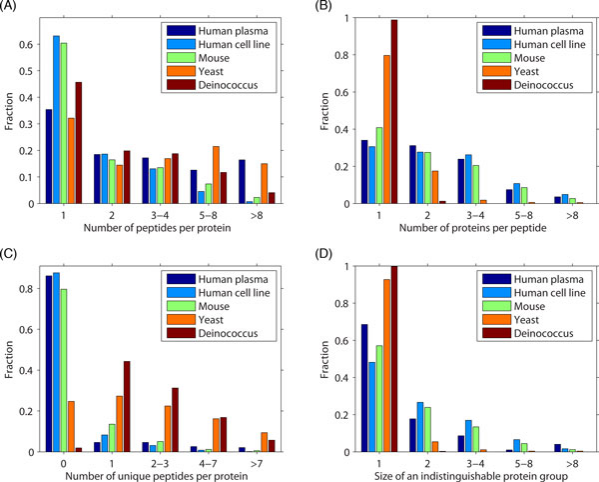


Figure 1A shows the percentage of identified peptides per protein for an FDR of 0.01 (on the unique peptide level) when using a reversed database as decoy. We observe that 32-63% of proteins are covered by only one confidently identified peptide, while 5-36% of proteins are covered by five peptides or more. Figure 1B shows the percentage of degenerate peptides in each sample. The results indicate that 57-68% of peptides in human and mouse samples are degenerate, regardless of the type of biological sample (e.g. cell line vs. tissue vs. plasma). On the other hand, the yeast and *D. radiodurans *data sets contain only 18% and 1% of degenerate peptides, respectively. Figure 1C provides the percentage of candidate proteins hit by unique peptides. In mouse and human samples more than 80% of candidate proteins are identified only with degenerate peptides. This percentage decreases to 23% for yeast and 3% for *D*. *radiodurans*. Finally, in Figure 1D we provide the percentage of protein groups of a particular size, where a group is formed from the set of proteins that are hit by exactly the same peptides. In accordance with previous results, most of the yeast and *D. radiodurans *candidate proteins are distinguishable; however, for human and mouse samples, between 30% and 50% of protein groups contain multiple proteins.

This analysis provides evidence that protein inference is a non-trivial problem, especially for multicellular eukaryotes that are known to contain large numbers of paralogous proteins. It also emphasizes the importance of developing sophisticated protein inference algorithms.

### Rule-based approaches

With a typical LC-MS/MS experiment resulting in a potentially large number of protein identifications, concerns were raised regarding the impact of misidentified proteins on biomedical science [45]. In response to this, several guidelines were proposed regarding the standards for publishing proteomics results [46-49]. The so-called "two-peptide rule" or two-hit rule, requiring two or more confidently identified peptides to define a confident protein identification, was advocated [46,48]. The same guidelines also recommended the parsimony principle (see next Section) as an explanation for the confident peptide identifications, and suggested that "protein family" - proteins with similar sequences due to single amino acid variants, homologs, splicing variants, or annotation mistakes - should be reported as one group if the proteins share the same identified peptides.

There is a good rationale for using the two-peptide rule. In principle, one correct unique peptide should be sufficient to correctly identify a protein. However, even for the low FDR associated with a set of peptides, many individual peptides in a large data set are incorrectly identified. Furthermore, proteins identified by single peptide hits are more likely to be incorrectly identified than proteins with higher peptide coverage [45]. It was reported that FDRs for single-hit proteins can be over 10 times higher than FDRs at the PSM level [50], likely due to the clustering of correct peptide identifications to the correct proteins and the lack of clustering behavior for the incorrect peptides [50,51].

However, the two-peptide rule has been challenged [51,52]. First, while including single-hit proteins without stringent quality control can compromise specificity, ignoring such proteins will certainly compromise sensitivity [52]. Second, controlling the confidence (FDR) at the peptide level and then deducing the proteins using heuristic rules leads to undefined FDRs at the protein level [27,50-52]. On the other hand, controlling FDR directly at the protein level may rescue some of the confident single-hit proteins. Indeed, Gupta and Pevnzer demonstrated that using the "single-peptide rule" results in 10-40% more protein identifications compared with the two-peptide rule at a fixed FDR level [52]. The single-peptide rule simply uses the highest scoring peptide from a protein as a score for that protein, and then directly estimates FDR at the protein level (rather than at the peptide level) using decoy databases. Thus, any protein that has one or more peptides with a score above a certain threshold is deemed confident. This statement seems problematic because proteins hit by single peptides should not be reliable. However, two mediocre peptides are not necessarily better than one good peptide; thus, many proteins hit by a single peptide can be rescued with more stringent score thresholds. Since a significant portion of such proteins are correct [53], it is not surprising that the single-peptide rule leads to more protein identifications.

With the help of protein-level FDR estimation (using a decoy database), better and more complex rules may be devised to achieve even higher sensitivity. For example, Weatherly et al. proposed setting separate score thresholds for proteins with different number of confident peptide identifications [51]. They reported that gradually lower score thresholds were needed for proteins with increasingly higher coverage. For the coverage of 1 (i.e. proteins hit by single peptides), a MASCOT score of 44 was required, while for coverage of 6, a MASCOT score as low as 11 was necessary for the same FDR [51].

Despite the relative simplicity of rule-based approaches, the performance of heuristic rules is fundamentally limited by the lack of rigorous treatment and proper combination of the peptide identification scores and prior knowledge.

### Combinatorial optimization algorithms

The input to this class of algorithms typically consists of a set of confidently identified peptides  = $\left\{p_{j}|t_{j}=1\right\}$ and a protein database . The objective is to provide a list of proteins that optimizes certain criteria. In one way or another, all such formulations result in NP-hard problems and are usually solved using approximation algorithms.

#### The minimum set cover formulation

**Figure 2:**
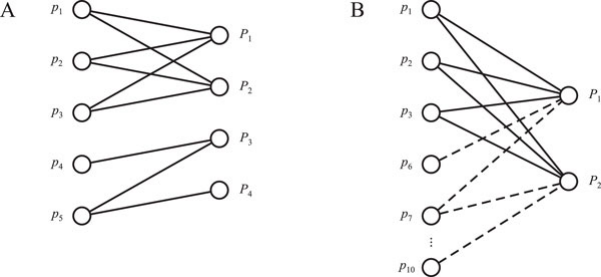


***Minimum set cover (MSC) problem: ***Given a set of confident peptide identifications  and protein data-base , find a smallest protein list L ⊆  such that each peptide from  is assigned to at least one protein from L. More formally,

This protein inference formulation is identical to the classical computer science problem of minimum set cover, where given a set of elements (peptides) U and a set of subsets (proteins) over U, the goal is to find a smallest (not necessarily unique) set of subsets that contain all elements in U. It is convenient to visualize the MSC formulation using bipartite graphs (Figure 2A). Using graph representation, it is relatively easy to see that an optimal solution to the MSC problem can also be provided if the original graph is divided into connected components and an optimal MSC solution provided separately for each component.

The MSC approach has been implemented in the IDPicker software [54,55]. IDPicker, however, also contains several heuristics that further simplify the solution and its interpretation. The algorithm starts by collapsing the peptide-protein bipartite graph such that all peptides/proteins connected to the same proteins/peptides form group nodes containing multiple peptides or proteins. It then finds a set of disconnected subgraphs within a bipartite graph using a depth-first search. Finally, it performs a MSC optimization in each of those subgraphs. IDPicker extends beyond algorithmic implementations, e.g. it contains modules for calculating confidently identified peptides (using an FDR-based approach), modules for combining scores from multiple search engines, as well as visualization of results.

The minimum set cover formulation is one of the most commonly encountered strategies in protein inference, and is recommended by the guidelines for publishing proteomics results [46,48]. Its intuition is to select the smallest among many possible solutions (Occam's razor, parsimony principle), which can be justified by considering the number of possible solutions when protein list consists of exactly  n proteins. Assuming  n ≪ ||, the solutions of smaller sizes are selected from a smaller solution space and are therefore less likely to be spurious findings. In many practical situations, including protein inference, the MSC formulation leads to natural and acceptable solutions. However, it is not obvious that a minimalist formulation should apply to biological samples in which multiple paralogous proteins or protein isoforms may be present at the same time. This approach also ignores other available information, e.g. peptides that are not identified (all dashed edges in Figure 2B), gene functions [56] or mRNA expression levels [57].

#### The partial set cover formulation

Although the MSC formulation relies on a set confidently identified peptides, a subset of such peptides are expected to be incorrect identifications. This fact provides motivation for the partial set cover approaches where the goal is to find the minimum protein list that covers at least 100·*c*% of the identified peptides, where 0 <*c *≤ 1 is a user specified parameter.

***Minimum partial set cover (MPSC) problem: ***Given a set of confident peptide identifications U, protein database , and parameter *c *(0 <*c *≤ 1), find a protein list L of minimal size such that at least 100·*c*% of identified peptides are assigned to the proteins from L. More formally,

where $z_{j}\in \left\{0,1\right\}$ indicates whether peptide  is excluded $\left(z_{j}=1\right)$ from the list of assigned peptides. Both MSC and MPSC problems are NP-hard in general. Thus, optimal solutions cannot be guaranteed in situations with a large number of identified peptides (note that each peptide from  adds a constraint in the problem formulation). A number of approximation algorithms have been proposed ranging from greedy algorithms to integer programming, and several such algorithms have been tested in protein inference [58].

Both the MSC and MPSC problem formulations result in situations where it is not possible to distinguish among proteins identified exclusively by degenerate peptides (e.g. proteins $P_{1}$ and $P_{2}$ in Figure 2). Nesvizhskii and Aebersold have identified several such classes of proteins, naming them indistinguishable proteins, subset proteins, subsumable proteins, etc. [20]. Because such situations are common for eukaryotes or samples containing multiple closely related organisms, different problem formulations are necessary to provide appropriate tie resolutions.

#### The minimum missed peptide formulation

The MSC-based formulations of the protein inference problem rely only on peptides that were confidently identified () and thus ignore all unidentified peptides from the proteins containing at least one peptide from , see dashed edges in Figure 2B. In addition, these methods implicitly assume that each peptide is equally likely to be observed in an MS/MS experiment. The first combinatorial approach addressing these aspects was the minimum missed peptide (MMP) formulation [59]. This approach relies on the concept of peptide detectability (Box 1).

**Figure 3:**
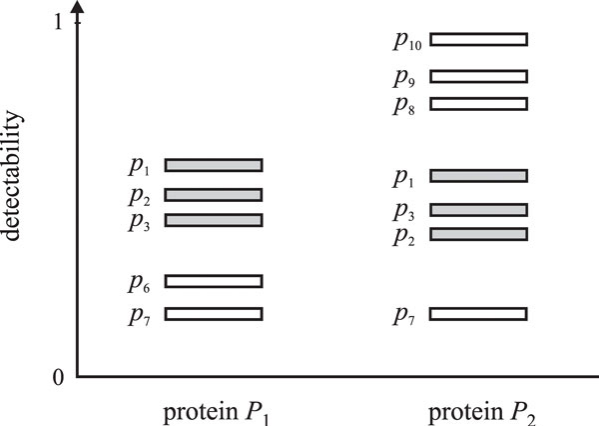


To provide intuition for the MMP approach, let us consider the example in Figure 3, which itself corresponds to the bipartite graph from Figure 2B. When considering only peptides in  (solid lines in Figure 2B), proteins $P_{1}$ and $P_{2}$ would be classified as indistinguishable [20]; however, given detectabilities of all peptides, it can be inferred that protein *P*_1 _is more likely to be present in the sample than protein *P*_2_. Specifically, the three identified peptides (shaded) are the most detectable peptides in protein *P*_1_. On the other hand, these peptides are among the least expected peptides to be observed if protein *P*_2 _was in the sample. Thus, protein *P*_1 _is more likely to be a correct identification than protein *P*_2_. Note that the tie resolution was provided by considering unidentified peptides.

Before formalizing the MMP approach, let us consider a particular *solution *to the protein inference problem in which different peptides from  are *assigned *to protein $P_{i}$. Note that some peptides  may not be assigned to $P_{i}(x_{ij}=0$ although their sequence can be mapped to the protein and the peptide is confidently identified $\left(t_{ij}=1\right)$. Peptide $p_{ij}$ is defined as *missed *if  and

where $d_{ij}$ is detectability of peptide $p_{ij}$. In other words, a peptide is missed if in a particular inference solution (1) it is not confidently identified and (2) a peptide with lower detectability from the same protein is identified and assigned to that protein. We emphasize that the peptides with detectabilities lower than the minimum detectability of assigned peptides for protein $P_{i}$ are not considered missed due to the fact that protein quantity influences effective detectability of all peptides in *P_i_*. Thus, for effective detectability below a certain threshold, no peptides are expected to be observed. The MMP approach can now be formalized as follows.

***Minimum missed peptide (MMP) problem: ***Given a set of confident peptide identifications , protein database , and peptide detectability for each peptide , find a set of proteins  that covers all peptides in  and minimizes the number of missed peptides. More formally,

$$\begin{array}{c} \text{minimize}\;\underset{i,j}{\sum }z_{ij}\cdot \left(1-t_{j}\right)\\ \text{subject}\;\text{to}\;\text{(}z_{ij}-z_{ik})\cdot \left(d_{ij}-d_{ik}\right)\geq 0\;\left(\forall i,j\in N\left(i\right),k\in N\left(i\right)\right)\\ \underset{i\in N\left(j\right)}{\sum }z_{ij}\geq t_{j}\;\left(\forall p_{j}\in C\right), \end{array}$$

where $z_{ij}\in \left\{0,1\right\}$ indicates whether detectability $d_{ij}$ for peptide  is above or equal to $\left(z_{ij}=1\right)$ or below $\left(z_{ij}=0\right)$ the minimum detectability of peptides assigned to protein $P_{i}$ and *N*(*i*) is a set of peptides connected to $P_{i}$ in the expanded bipartite graph (see Figure 2B). A set of identified proteins can now be determined as

$$y_{i}=\left\{\begin{array}{c} 0\;\text{if}\sum_{j\in N\left(i\right)}z_{ij}\cdot t_{j}=0\\ 1\;\text{if}\sum_{j\in N\left(i\right)}z_{ij}\cdot t_{j}>0 \end{array}\right)$$

Alves et al. have shown that the minimum cover set problem can be reduced to the minimum missed peptide formulation [59]. Thus, the MMP problem is NP-hard and approximation algorithms are needed for large-scale problems. Alves et al. proposed an efficient greedy approximation algorithm that provides a good solution [59-61]. Alternative formulations and algorithmic approaches are also possible. For example, this algorithm can be generalized in a relatively straightforward manner to a partial set formulation or to a version that minimizes the overall probability of unidentified peptides.

Although the MMP formulation was the first protein inference technique capable of resolving indistinguishable proteins, it generally shares the limitations of other approaches based on combinatorial optimization techniques. That is, these algorithms do not provide probabilities for identified proteins, unless post-processing statistical models are used [62].

### Probabilistic inference algorithms

Similarly to the previous classes of algorithms, probabilistic approaches to protein inference generally consist of two steps. First, PSM scores are converted to PSM probabilities using algorithms such as PeptideProphet [38]. After this pre-processing step, protein inference is performed based on an assumed probabilistic model. In probabilistic terms, protein inference involves computing protein posterior probabilities  for every protein in .

Several classes of probabilistic algorithms have been proposed so far [21,24,60,61,63-71], with different strategies and levels of rigor in addressing protein groups and different run-time performance. Some probabilistic algorithms do not address degenerate peptides [63,65,68,70], while some such as ProteinProphet [21] combine probabilistic inference with the parsimony principle (for degenerate peptides) and protein grouping (for indistinguishable proteins). In the following subsections, we provide an in-depth discussion of the three major probabilistic methods: ProteinProphet [21], MSBayesPro [61], and Fido [71], and briefly mention several other methods. We use the same notation for all models and, when possible, provide new interpretations of the algorithms. We aim to reveal inherent connections and principal differences among the methods. For original derivations and interpretations, readers are referred to the original publications.

#### ProteinProphet

ProteinProphet is the first and most widely used probabilistic protein inference approach [21], with importance comparable to the first automated peptide identification tool, SEQUEST [9]. ProteinProphet consists of four major steps; together, they convert the original PSM probabilities from PeptideProphet to peptide identification probabilities and then combine the peptide identification probabilities to infer proteins.

##### Pre-processing

**Figure 4:**
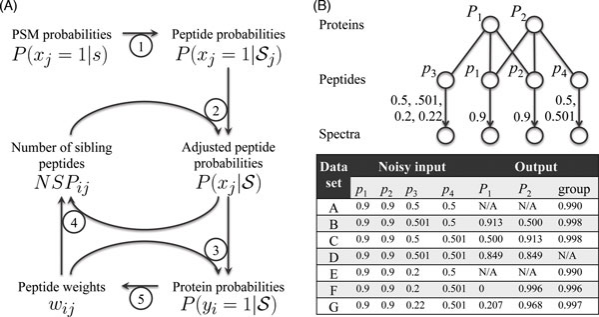


In order to obtain protein identification probabilities, peptide identification probabilities are needed as input. Here, the difficulty is to obtain one peptide identification probability from typically multiple spectra matched to a peptide. The solution used in ProteinProphet is to simply take the maximum value among the peptide-spectrum matching probabilities for peptide  j (step 1, Figure 4A), i.e.

where  is the set of spectra identified for peptide  j. If no spectrum is matched to the peptide, i.e. if  then . Recently, the iProphet algorithm was proposed to improve this approach [72].

##### Combining peptide probabilities

A key feature of ProteinProphet is that protein probabilities are computed by assuming peptide identifications to be independent pieces of evidence for the presence of protein  i in the sample, i.e.

where N(i) is the set of peptides mapped to protein  i. This assumption, however, is not easy to justify because peptide identifications are not statistically independent. That is, if one peptide from the protein is confidently identified, the chance is higher that another peptide from the same protein will also be identified. Another problem with this assumption is that each degenerate peptide is counted toward all proteins it maps to. These issues are addressed via the following two adjustment steps.

##### Adjustment for peptide identification probability

To address the limitation due to the independence assumption, ProteinProphet replaces  in the equation above by ; step 2, Figure 4A. The difference between the adjusted peptide identification probability ) and the original peptide identification probability  comes from the presence of other spectra (peptides) mapped to the same protein as peptide  j. They are expected to change the confidence of peptide identification. However, it is not straightforward to estimate . Nesvizhskii et al. defined the expected number of sibling peptides (NSP), i.e. the number identified peptides (other than peptide $p_{j}$) weighted by the adjusted peptide identification probability ), from the same protein. Specifically, $NSP_{ij}=\sum_{j^{\prime }\in N\left(i\right),j^{\prime }\neq j}P\left(x_{j^{\prime }}=1|S\right)$, where  i indexes a parent protein of peptide  j (step 4, Figure 4A). ProteinProphet then approximates , which is computed from ) and ) by using the Bayes rule. Since computing *NSP_ij _*requires , and computing  requires *NSP_ij_*, iterative updating is used until convergence (steps 2, 4; Figure 4A).

##### Adjustment for peptide degeneracy

In order to address degenerate peptides, a weighting scheme is used to modify protein probabilities to

$$P\left(y_{i}=1|S\right)=1-\underset{j\in N\left(i\right)}{\prod }\left(1-w_{ij}\cdot P\left(x_{j}=1|S\right)\right),$$

where *w_ij _*is the "proportion" of peptide *j *assigned to protein *i *(step 3, Figure 4A). Nesvizhskii et al. defined that $w_{ij}=P\left(y_{i}=1|S\right)/\sum_{i^{\prime }\in N\left(j\right)}P\left(y_{i^{\prime }}=\;1|S\right)$, where *N*(*j*) is the set of proteins that contain peptide  (step 5, Figure 4A). This adjustment step is in accordance with the parsimony principle cause $\sum_{i\in N\left(j\right)}w_{ij}=1$, i.e. one peptide is ensured to come from only one protein in total. Note that *w_ij _*= 1 for all unique peptides and that *w_ij _*= 0 if peptide *j *cannot be mapped to protein *i*, i.e. when i∉N(j). Since the calculations of *w_ij _*and  are mutually dependent, another iterative updating procedure is used until convergence.

By combining these four steps, with a minor modification to include weights *w_ij _*for peptides in the NSP adjustment step, i.e. $NSP_{ij}=\sum_{j^{\prime }\in N\left(i\right),j^{\prime }\neq j}w_{ij}\cdot P\left(x_{j^{\prime }}=1|S\right)$, protein identification probability  can be approximated through a variant of the expectation-maximization (EM) iterative process (steps 2-5; Figure 4A). Since indistinguishable proteins remain indistinguishable in ProteinProphet, the grouping strategy is adopted by treating the indistinguishable proteins as one protein. Therefore, a "group probability", i.e. the probability that any one of the proteins in the group is identified, is reported.

As the first probabilistic inference method for protein identification, ProteinProphet has been very successful and, as part of the Trans-Proteomic Pipeline [73], remains the most widely used protein inference tool. Although the degenerate peptides are handled by a parsimony-driven weighting procedure, an iterative method by ProteinProphet is used to obtain those weights and ultimately results in reasonable probabilities for proteins. Recently, the tool has been improved, mainly at the pre-processing step, due to iProphet [72]. By using the same computational strategy as in the NSP adjustment step of ProteinProphet, iProphet obtains one identification probability for each peptide by aggregating the PSM probabilities of the peptide from multiple search engines, spectra, experiments, charge states, and PTM states.

##### Limitations

Because ProteinProphet relies on certain strong assumptions, e.g. the parsimony-driven weighting (step 5, Figure 4A), its outputs are not always sensible from a statistical perspective. One such scenario was noticed by the authors [21], that for a set of proteins with shared peptides, a protein with a unique peptide, no matter how small the identification probability is, always dominates the protein(s) without unique peptides. In other words, the algorithm assigns score 1 to the protein with a random but unique peptide identification and score 0 to other proteins. This is undesirable, since there are always a large number of random peptide identifications with close to 0 probabilities in real proteomics data sets. To address the issue, only peptides with probabilities ≥0.2 are used to compute protein probabilities. Similarly, we observed that the inference outcome of ProteinProphet is sensitive to minor changes in peptide probabilities. This can be illustrated by a simple example shown in Figure 4B. Consider two homologous proteins *P*_1 _and *P*_2 _with identified peptides {*p*_1_, *p*_2_, *p*_3_} and {*p*_1_, *p*_2_, *p*_4_}, respectively. Suppose *p*_1 _and *p*_2 _are reliable identifications, but that *p*_3 _and *p*_4 _are not, with small identification probabilities. In the seven toy datasets (A-G) in Figure 4B, we varied the identification probability of peptides *p*_3 _and *p*_4_, and computed the protein probability using ProteinProphet. In data sets A and E, when the probabilities of unique peptides are not larger than 0.5, ProteinProphet considers proteins *P*_1 _and *P*_2 _indistinguishable, and only reports a group probability; in data set B, when probability of peptide *p*_3 _is slightly larger than *p*_4 _(which has probability 0.5 or less), ProteinProphet considers protein *P*_1 _as much more reliable than *P*_2_; in data sets C and G, when probability of peptide is (slightly) larger than *p*_3 _(which has probability 0.5 or less), ProteinProphet considers protein *P*_2 _as much more reliable than *P*_1_; in data set D, when the probabilities are both larger than 0.5, ProteinProphet considers both proteins to be reliable; while in data set F, when the probability of peptide *p*_3 _is 0.2 or less, ProteinProphet suggests that only protein *P*_2 _can be the true protein, despite the significant probability that peptide *p*_4 _is a random identification. This non-continuity of the inference results is counterintuitive. Naturally, one would expect the probability of protein *P*_2 _(*P*_1_) decreases (increases) gradually as the probability of peptide *p*_3 _decreases.

Although ProteinProphet applies the parsimony principle to the issue of shared peptides, it uses a probabilistic model and an EM-like algorithm. Thus, ProteinProphet distinguishes itself from the other parsimony principle-driven methods, such as the combinatorial approaches discussed earlier. However, it is not clear how often ProteinProphet actually leads to the same solutions as other various combinatorial approaches regarding proteins with shared peptides. In addition, with the presence of degenerate peptides, the inference problem is difficult; thus, it would be interesting to compare the EM-like iterative algorithm used by ProteinProphet with the heuristics used by the combinatorial approaches to examine how efficiently they handle large data sets.

#### MSBayesPro

MSBayesPro [61] is defined as a full probabilistic protein inference method which provides "perhaps the most rigorous existing treatment of the peptide degeneracy problem" [71]. The MSBayesPro model includes peptide detectability in the probabilistic model; thus it can, to some degree, distinguish among "indistinguishable" proteins.

##### Model structure

**Figure 5:**
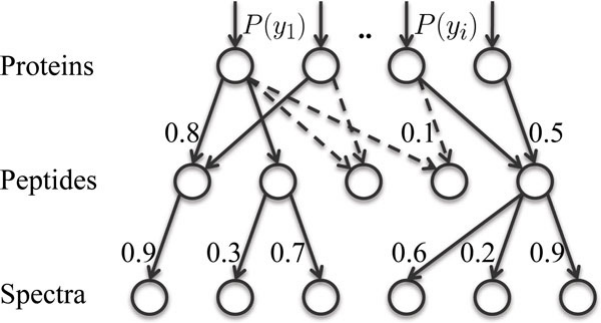

MSBayesPro is a Bayesian network (Figure 5) serving as a generative model for the data. The high level structure of the network is simple: Proteins → Peptides → Spectra, which mimics the experimental protocol in proteomics where proteins are first digested into peptides, from which spectra are generated. Hence,

P(y,x,S)=P(y)P(x|y)P(S|x)∝P(y)P(x|y)P(x|S),

where *y *is a vector of random indicator variables for all candidate proteins, *x *is a vector of random indicator variables representing *all *peptides from those proteins, and S represents the data, i.e. all the spectra generated in the experiment. The Peptides → Spectra associations are defined by the available PSM scores (or probabilities). The Proteins → Peptides connections, however, are determined by the sequences of the peptides and candidate proteins. If the sequence of peptide *p_j _*can be exactly mapped to protein *P_i_*, there will be an edge pointing from the protein node *i *to peptide node *j *in the network. This is similar to the structure of the model used in ProteinProphet, although the latter is not a Bayesian network. However, there is an important difference between MSBayesPro and ProteinProphet, i.e. all peptides, identified and unidentified, are included in the network structure in MSBayesPro. In contrast, the unidentified peptides are ignored in ProteinProphet and other Bayesian network models [69,71] proposed subsequently. Other than the simplification of the model structure, we believe there is no legitimate justification for excluding unidentified peptides from a probabilistic model. Such peptides will have the identification probability ; thus *x_j _*= 0 is guaranteed in the inference step. We note that it is these unidentified peptides that, together with the peptide detectability information, will lead to tie resolution between grouped proteins and improve the scoring of proteins hit by single peptides.

The MSBayesPro model has an important property in that the peptide identifications are conditionally independent given the presence of the parent proteins (Figure 5). This is not to be confused with the independence assumption of peptide identification used in ProteinProphet. Actually, the conditional independence assumption in MSBayesPro will lead to marginally dependent peptide identifications if two peptides share parent proteins directly or indirectly through other peptide/protein nodes (that is, if the two peptides are in a connected component of the graph). Furthermore, the conditional independence assumption aligns with the LC-MS/MS experiment. Consider a protein $P_{i}$ that is in the sample at some known abundance *q_i_*. Then, further knowing the information that one peptide is already identified from this protein does not inform whether another peptide from the same protein should be identified in MS/MS or not. With conditional independence, we can expand the joint probabilities of the set of peptides *N*(*i*) (both the identified ones and those that are not) from protein *i *as

$$P\left(x_{N\left(i\right)}|y_{i}=1,y_{i^{\prime }\neq i}=0,q_{i}=q\right)=\prod_{j\in N\left(i\right)}P\left(x_{j}|y_{i}=1,y_{i^{\prime }\neq i}=0,q_{i}=q\right).$$

where *q_i _*is the abundance of protein *P_i_*.

##### Model inputs and parameters

MSBayesPro requires peptide identification likelihood ratios and a set of peptide detectabilities. The former is a required input to the method, and the latter, as required parameters of MSBayesPro, can be provided as an input, or ideally, peptide detectabilities should be estimated via a machine learning model from the same data set on which protein inference is carried out [24,61].

For peptide identifications, the input to MSBayesPro is the likelihood ratios  rather than the peptide identification probabilities  that implicitly include a uniform prior [60,61]. Here the original peptide-invariant class priors used to compute peptide identification probability are replaced in MSBayesPro by the peptide sequence and protein abundance dependent detectabilities, which are more informative priors. We note that this treatment in MSBayesPro is somewhat related to the NSP adjustment in ProteinProphet, which essentially changes the prior to incorporate information from the NSP values (interestingly, NSP values may roughly reflect protein abundances, in similar ways as effective detectability). Note that unlike detectability, NSP is not specific to the sequence of a peptide.

Using peptide detectability is an important distinguishing feature of MSBayesPro. Detectability is required to build the conditional distribution tables between the Protein and Peptide layers and subsequently to compute the posterior probabilities for the proteins. However, to use detectability properly it is important to consider the impact from protein quantity (Box 1). Li et al. [60] proposed a quantity adjustment formula to convert *standard peptide detectability *$d_{ij}^{0}=P\left(x_{j}=1|y_{i}=1,\;\;q_{i}=q^{0}\right)$ to *effective detectability *$d_{ij}\left(q\right)=P\left(x_{j}=1|y_{i}=1,q_{i}=q\right)$, where *q_i_*, the quantity of protein *P_i_*, is estimated by the maximum likelihood or moment matching approaches. If a (degenerate) peptide *p_j _*is shared by multiple proteins, the network structure requires combining detectabilities *d_ij _*over all parent proteins of *p_j_*. Here, MSBayesPro assumes that $d_{j}=1-\prod_{i\in N\left(j\right)}P\left(x_{j}=0|y_{i}=1,\;\;q_{i}\right)=1-\prod_{i\in N\left(j\right)}\left(1-d_{ij}\right)$. Alternative approaches in combining multiple detectabilities may also work, but the key intuition is the following: if, for a given peptide, there are multiple parent proteins all present in the sample, the detectability of the peptide should be larger than its detectability from any of the individual proteins alone. This treatment permits a non-parsimonious solution, because a degenerate peptide is allowed to come from more than one parent protein.

##### Inference algorithms

With the Bayesian network model structure and parameters specified, it is in principle easy to exactly compute the joint posterior probability for the proteins, i.e. . An optimal solution for the presence of all proteins (the maximum *a posteriori *configuration) is computed as . The joint posterior probability can be further marginalized to compute  for the presence of each individual protein in the sample. In practice, this is not always possible due to the prohibitive time complexity, i.e. the inference on Bayesian networks is NP-hard in general [74]. MSBayesPro uses Gibbs sampling instead of exact computation when a connected component in the Bayesian network is large (it is easy to show that connected components should be considered separately).

It is important to note that MSBayesPro also reports estimated protein quantities and the marginal posterior probabilities for peptides, which provide better scores for measuring peptide confidence [61]. Thus, in its core, MSBayesPro is also a label-free quantification algorithm. Further generalization of the MSBayesPro model has been suggested to unify the peptide and protein identification problems and perform higher-level inference on genes and pathways based on proteomics data [75].

##### Limitations

The use of peptide detectability is both the strength and a limitation of MSBayesPro. The method requires good detectability predictions in order to achieve good performance [24]. However, prediction of detectability for non-tryptic peptides and post-translationally modified peptides is not a fully solved problem yet, which limits the applicability of MSBayesPro. In addition, detectabilities cannot be expected to provide tie resolution for proteins with nearly identical sequences. These cases, however, reveal the limits of shotgun proteomics experiments and should be addressed by follow-up experiments such as well-designed targeted proteomics experiments. Another limitation is related to the computational complexity: efficient approximation algorithms are necessary for MSBayesPro to work on very large data sets.

#### The Fido model

The Fido model [71,76] uses a Bayesian network, but was primarily designed for fast inference. The major contribution of this method consists of two graph transformations applied to each connected component: collapsing protein nodes that are connected to the identical sets of peptides and pruning of spectral nodes (with user specified parameters) that results in splitting of the connected components. Both transformations facilitate tradeoffs between the accuracy and speed of the inference step. Fido also allows an application of advanced probabilistic inference algorithms, e.g. the junction tree algorithm, which significantly improve protein inference on large graphs.

**Figure 6:**
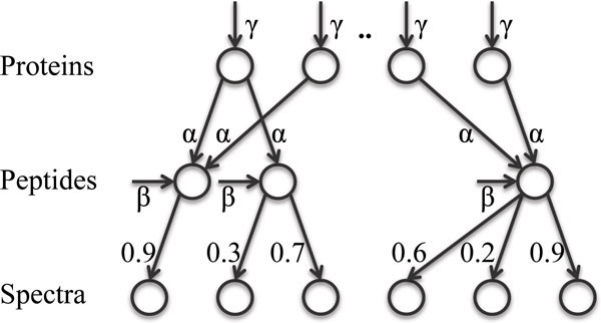


There are two major differences in the Bayesian network models used by Fido and MSBayesPro. First, unidentified peptides are ignored in Fido and a sequence-independent parameter is used as a replacement for peptide detectability (Figure 6). Hence, the resulting Bayesian network is simpler and inference is faster. Second, another parameter *β *is introduced to the model, which is the prior probability for a peptide to be identified from an artificial "noise" node. This addresses the situation where input peptide probabilities are not accurate (e.g. many incorrect peptides are assigned high probability). We believe this is a legitimate remedy for disasters that can happen during the peptide probability estimation. However, parameter *β *seems to be redundant given that ${\left(1-\alpha \right)}^{\left|N\left(j\right)\right|}$ is the probability for a peptide *p_j _*to be identified from "noise". The authors indeed observed strong inverse correlation between the optimal values of *α *and *β*.

One limitation of the Fido model is that it requires a decoy (randomized) database to find the best values of the parameters ( α,  β, and  γ- the prior for the presence of proteins) by combining an ROC optimization (in a supervised manner) with FDR estimation. Some versions of this approach may lead to overly optimistic performance estimates. Decoy database-independent maximum likelihood approach may be an alternative to fit the parameters. Finally, the parameter optimization step dramatically increases the run time of the algorithm (up to 2000 times), which compromises the overall speed of the method [71].

#### Other probabilistic approaches

Yang et al. recently investigated protein inference from an information retrieval (IR) point of view [68]. This work is interesting because it leverages methods in the IR field to the protein identification problem in proteomics. The authors found that the Prob-OR score, which is similar to ProteinProphet without the two adjustment steps, is dramatically worse than Prob-AND score, which is related to the protein posterior probabilities computed by MSBayesPro if degenerate peptides were treated as unique to each parent protein. We emphasize that the IR method proposed by Yang et al. is inherently a ranking approach rather than an inference approach; hence, it does not directly address the shared peptide issue as do the other probabilistic approaches discussed above.

Gerster et al. [69] recently reported a new probabilistic approach, Markovian Inference of Proteins and Gene Models (MIPGEM), that is similar to MSBayesPro and Fido. MIPGEM models peptide probabilities as random variables as in some previous approaches [66] and assumes conditional independence between peptide scores given their parent proteins (Markovian assumption). Similar to the Fido model, MIPGEM does not consider peptide detectability or unidentified peptides although the authors suggested that including detectability would be a future consideration. Table 2 provides a summary of the major probabilistic inference methods. Several other methods are reviewed in [35-37].

**Table 2:** A comparison between different probabilistic protein inference algorithms.

Methods	ProteinProphet	MSBayesPro	Fido	MIPGEM
Underlying graph structure	Bipartite graph with identified peptides and matching proteins^1^	Bayesian network with all peptides from proteins with at least one identified peptide	Bayesian network with identified peptides and matching proteins	k-partite graph with identified peptides, matching proteins and (optionally) matching gene models^2^

Inference algorithm	EM (Expectation Maximization) like	1) Exact^3^; 2) Memorizing-Gibbs sampling	1) Exact^3^ ; 2) Pruning approximation	1) Exact^3^; 2) Direct sampling

Input	Probabilities for peptides with user-defined cutoff for *p *(often *p *> 0.05 is used)	Likelihood ratios for peptides with *p *> 0.05 and peptide detectabilities	Likelihood ratios for peptides with *p *> 0.05	Probabilities for peptides with user-defined cutoff for *p *(often *p *> 0.05 is used; 0.9 for best performance)

Output	1) Protein probabilities; 2) Protein group probabilities; 3) NSP adjusted peptide probabilities	1) MAP solution, protein abundances and probabilities; 2) Protein group probabilities; 3) Posterior peptide probabilities	1) Protein probabilities; 2) Protein group probabilities	1) Protein probabilities; 2) Gene model probabilities

Protein prior estimation	No protein priors	Direct frequency estimation based on protein posterior probabilities in one run of MSBayesPro	Grid search optimizing cross- validation performance through multi-runs of Fido with different priors	Grid search optimizing model likelihood through multi-runs of the MIPGEM with different priors

Peptide probability adjustment by	NSP from a parent protein	Protein quantity adjusted peptide detectability	Two detectability-like parameters *α*, *β*	Treating peptide identifications as random variables

Protein grouping	Yes	No (indistinguishable proteins are resolved)	Yes	No (indistinguishable proteins are not resolved)

Peptide charge	Considered	Ignored	Considered	Considered

Novel aspects	1) First probabilistic protein inference algorithm; 2) Efficient EM algorithm	1) A Bayesian network; 2) Resolves indistinguishable proteins using unidentified peptides and peptide detectability; 3) Modified Gibbs sampling	1) Using a noise model to remedy inaccurate peptide probabilities; 2) Pruning algorithm, efficient inference	Gene model probabilities^4^

Availability	http://tools.proteomecenter.org	http://darwin.informatics.indiana.edu/yonli/	http://noble.gs.washington.edu/proj/fido	-

1. For ProteinProphet, the underlying bipartite graph does not correspond to a Bayesian Network although it guides the EM-like algorithm through inference.

2. MIPGEM uses a rule-based protein removal scheme to simplify the network structure;

3. Exact computation is used only for small connected components;

4. Gene centric proteomics was proposed in [77], and implemented earlier in a deterministic way in [67].

## Discussion

Our main goal in this review was to present the challenges, intuition and proposed solutions to the protein inference problem. With increased throughput of proteomics experiments, the tools and approaches presented here will have increasingly more important applications to many problems in biology and biomedical sciences. These applications include inference and verification of gene models, identification of splice forms or post-translatioinally modified sites. Some of these problems can only be addressed using proteomics techniques and, as such, proteomics holds great promise in systems biology, biomarker discovery, diagnostics, prognostics and treatment monitoring.

Undoubtedly, there is a need for more sophisticated methodology for protein inference, unbiased performance evaluation of these techniques, as well as stand-alone tools with graphical user interface that will facilitate transition from research environments to practice in biomedical sciences. We conclude this paper by discussing the current issues in evaluating protein inference algorithms and then speculating on the ideal protein inference approaches.

### Evaluation of protein identification methods

Despite the development of computational protein identification methods, objectively evaluating the performance of the methods remains a problem. Two strategies are currently available: the use of standard samples (mixtures of known proteins) and the use of decoy protein sequences to estimate FDR at the protein level. Both approaches have limitations.

To date, only a limited number of standard samples [78-80] containing 10-50 proteins have been used to facilitate evaluation of peptide/protein identification. The advantage of using standard samples is that the truth is known; thus, the accuracy measures, e.g. precision and recall, of protein identification can be directly computed. However, standard samples are frequently plagued by contaminant proteins and the boundary between true and false protein identification is blurred. Another limitation of standard samples is their small number of proteins, which leads to difficulties in assessing statistical significance in method comparisons.

The second approach estimates protein-level false discovery rates with the help of decoy databases. Although the approach has been used in several studies [51,52], two serious problems of the approach are generally ignored. We suggest that using decoy databases for evaluation of protein identification algorithms should be approached with these limitations in mind. First, unlike the decoy (e.g. reversed, randomized) database approach for peptides, the decoy database for proteins does not produce the correct estimation of the number of incorrect protein identifications when the correct proteins comprise a significant portion of the database. In an extreme scenario, when all proteins in the database are present in the sample, all the identified proteins from the forward database are correct despite many peptides being in-correct identifications. On the other hand, all identified proteins from a decoy database are incorrect. Thus, using a decoy directly will produce a non-zero FDR, while FDR = 0 is the correct answer.

This problem can be addressed by correcting for the bias due to the number of true proteins in the forward database. Let the number of identified forward and decoy proteins be *n_F _*and *n_D_*, and the total number of forward and decoy proteins in the databases be *N_F _*and *N_D_*, respectively. Let the protein level FDR in forward database be *FDR_P _*and the rate of incorrect protein identifications from the forward and decoy database be

$$\gamma_{F}=\frac{FDR_{P}\cdot n_{F}}{N_{F}-\left(1-FDR_{P}\right)\cdot n_{F}},$$

and

$$\gamma_{D}=\frac{n_{D}}{N_{D}},$$

respectively. An assumption regarding a decoy database is that the rates of the false protein identifications are identical; hence, $\gamma_{F}=\gamma_{D}$. By solving this equation we find

$$FDR_{P}=\frac{n_{D}\cdot \left(N_{F}-n_{F}\right)}{n_{F}\cdot \left(N_{D}-n_{D}\right)}.$$

Note that there is a correction factor $\left(N_{F}-n_{F})(N_{D}-n_{D}\right)$ in this equation compared to the FDR formula used for peptides. Also, when $N_{F}=n_{F},\;FDR_{P}=0$ as expected. A related correction is implemented in the MAYU approach [50] developed for FDR estimation from large proteomics data sets, i.e. the case when $n_{F}/N_{F}\gg 0$. Further corrections may be needed if the average lengths of the identified vs. non-identified proteins are different.

We would like to point out that, for probabilistic protein inference algorithms, theoretical protein FDR values can be computed based on the protein posterior probabilities. However, such theoretical FDR values are only accurate when the reported protein posterior probabilities are accurate. Hence, they need to be evaluated themselves, e.g. against the target/decoy-based empirical FDRs.

The second and more serious issue for applying the decoy approach is related to the existence of protein families. In fact, to our knowledge, no solution has yet been proposed. Simply speaking, a randomized database cannot serve as a good decoy for evaluating methods on data sets that contain many degenerate peptide identifications. The reason is that such peptides are typically shared among forward proteins, which could be similar to each other due to biological/annotation reasons, but not with decoy proteins. As a result, a randomized protein database cannot provide indications whether the identifications made among homologous proteins are correct or not. For this reason, a randomized decoy database is expected to underestimate FDRs for eukaryotic samples, which have large number of shared peptides (Figure 1). The problem might be addressed using well-constructed non-random sequence database or using a closely related proteome database as decoy. Evaluating protein inference algorithms using such non-random decoys, however, remains a research problem.

We emphasize that both standard mixtures and the target/decoy approach for complex samples have their pros and cons in evaluating protein inference algorithms, and they are not mutually exclusive approaches. In fact, standard mixtures can be used to validate the target/decoy approach for protein FDR estimation. It is generally a good idea to use both strategies for a more complete and objective evaluation.

#### A need for guidelines for comparisons between methods

Due to the complexity of protein inference, fair evaluation of the proposed methods has been challenging. This is due to two major aspects. First, reliable and objective validation of the protein identification results is itself a challenging problem, as the FDR estimation is still unreliable. In addition, it is not even obvious how to compare models whose outputs are considerably different, e.g. those that provide protein groups and those that resolve ties between all proteins. Second, due to the lack of agreed upon guidelines, avoidable unfair comparisons are sometimes seen in the literature [69]. In other works, different peptide identification algorithms or scoring schemes are sometimes used as inputs to different protein inference methods, making the protein inference comparisons uninterpretable.

In order to address this situation, we tentatively propose the following principles for comparisons of protein inference algorithms. First, whenever possible, the same or equivalent peptide identification scores as input to different programs should be used. Second, effort should be made to provide inputs most appropriate to each algorithm considered. For example, algorithms that take all peptide identifications should be provided all scores, while programs that take only confident identifications should be provided such a subset. Third, at least one standard protein mixture data set should be used and all known proteins (whether they belong to "indistinguishable" protein groups or not) in such data sets should be included in the evaluation of the protein inference methods. This will allow the evaluation of protein inference algorithms on proteins identified without any unique peptides. Finally, and in an ideal scenario, large data sets from complex samples of unknown proteins should also be used to compare different programs; however, we caution that the current decoy database strategy may not provide reliable FDR estimates at the protein level (evaluation for protein data sets with significant fraction of degenerate peptides is a particular problem).

### The ultimate protein inference approach

Despite the amount of published work, the protein inference problem is far from solved. We believe two aspects are crucial to the future approaches. First, the model should be probabilistic and with degenerate peptides treated in principled ways. Second, unidentified peptides should be exploited with peptide detectability incorporated into the model, perhaps adjusted to allow modeling peptide competition at the elution stage in a given sample. Despite the current limitations of peptide detectability predictions, especially for non-tryptic and modified peptides, it is believed that including detectability [24,35,69,71] or peptide-specific information for peptide probability adjustment [21] would improve the current methods for protein inference.

Furthermore, we believe that better estimation of peptide/protein quantity might also help protein inference by, for example, improving the quantity adjustment of peptide detectability [60,61], and provide additional input information for protein inference. As mentioned in the Introduction, protein inference can be viewed as a special case of protein label-free quantification. In fact, an ideal inference algorithm should automatically be a quantification algorithm, and vice versa. We believe much better performance can be achieved by combining the protein inference and quantification tasks into one statistical framework.

Algorithmic development is equally important for rigorous and yet practical probabilistic inference. Serang et al. [76] proposed an approximate solution by setting low peptide probabilities to zero and then applying the graph pruning procedure. In this way the complexity of the problem can be controlled at arbitrarily low levels with the price of potentially high error (i.e. the computed probability may greatly deviate from the exact values). The Gibbs sampling approach implemented in MSBayesPro can achieve arbitrarily high accuracy in probability estimation; however, the time required for the inference can be prohibitively long. A fast algorithm with controllable error bound is desirable. Applying well-established exact or approximate graph inference algorithms, e.g. the junction tree algorithm [76], is an important direction for further investigation.

## Competing interests

The authors declare that they have no competing interests.

## Supplementary Material

Additional file 1 Peptide detectability. Click here for file
