# Supplementary material for: Computational approaches to protein inference in shotgun proteomics
Source: BMC Bioinformatics. 2012 Nov 5;13(Suppl 16):S4. doi: 10.1186/1471-2105-13-S16-S4 (PMC3489551; doi:10.1186/1471-2105-13-S16-S4)
Supplement: Additional file 1 — Peptide detectability. [file 1471-2105-13-S16-S4-S1.pdf]

Peptide detectability

Peptide detectability has been defined as the probability that a peptide will be identified in a proteomics experiment given the presence of its parent protein in a sample [19, 24]. There are multiple factors, spanning all phases of a proteomics experiment, that influence peptide identification. For example, during sample storage and preparation, some peptides may be truncated at their termini resulting in semi-tryptic or non-tryptic peptides (in the case of trypsin digestion) which usually remain unidentified in a database search [25]. Peptides with different hydrophobicity patterns may not be retained in the LC stationary phase (hydrophilic peptides) or may be insoluble in the LC mobile phase (hydrophobic peptides). Peptides that eluted will be ionized with different efficiencies based on the presence and distribution of charged residues in their sequence. Furthermore, in complex biological samples, peptides are likely to co-elute with many other peptides and thus compete for ionizing protons during the electrospray ionization. Many peptides may elute and ionize well, but poorly fragment, producing MS/MS spectra with few peaks. Such peptides are difficult to interpret by computational methods. In addition, peptides whose *m/z* values are outside of the range of the mass spectrometer (200-2000 Da) cannot be identified. Apart from physicochemical aspects, there are several biological factors influencing peptide identification. For example, the three-dimensional structure of a protein could lead to the existence of sites with different sensitivities to proteolytic digestion. Other sites may be post-translationally modified by one of more than 200 different post-translational modifications (PTMs) observed in eukaryotes [26]. Many such peptides typically remain unidentified unless a database search explicitly specifies the PTM type or the sites of interest (which, in turn, leads to a decrease in the number of identifications for regular peptides). Finally, different peptide identification software packages are based on different assumptions and are known to result in differences among identified peptides [27].

It has been shown that the detectability of a peptide at standard quantity, i.e. *standard detectability*, is a property of the peptide sequence and thus can be predicted from peptide/protein sequence for a given experimental platform [17, 19, 24, 28-33]. On the other hand, the quantity of a protein also determines the fate of a peptide with respect to its identification. For example, peptides with high standard detectability that are present in a sample in low quantity may not be identified, while peptides with relatively low detectability present at high quantity may in fact be observed. Therefore, protein quantity and standard detectability collectively determine the *effective detectability* of each peptide in a protein. Effective peptide detectability cannot be predicted from amino acid sequence alone (unless protein quantity can be shown to depend on protein sequence) and has to be estimated from a set of peptide identifications and their standard detectabilities [24].

A detectable peptide is related to a *proteotypic peptide*, which is “an experimentally observable peptide that uniquely identifies a specific protein or protein isoform” [18]. In practice, proteotypic peptides were required to be observed in more than 50% of experiments in which their parent protein was identified [18, 30]. The relationship between two definitions can be best understood from an interesting property of peptides in MS/MS experiments to group at either high or low end of the detectability scale in a standard sample [24, 34].

In summary, the identification of a peptide in a proteomics experiment is a stochastic event that depends on multiple factors. It is therefore convenient to summarize all these factors using a probabilistic frame-

work. The standard detectability of peptide p_j from protein P_i (at quantity q^0) will be denoted as d_{ij}^0, while the effective detectability at an arbitrary quantity q will be denoted as $d_{ij}(q)$.
